# Supplementary material for: Multiple valence bands convergence and strong phonon scattering lead to high thermoelectric performance in p-type PbSe
Source: Nat Commun. 2022 Jul 19;13:4179. doi: 10.1038/s41467-022-31939-4 (PMC9296461; doi:10.1038/s41467-022-31939-4)
Supplement: Supplementary file 1 — Supplementary Information [file 41467_2022_31939_MOESM1_ESM.pdf]

Supplementary Information

Multiple valence bands convergence and strong phonon scattering lead to high thermoelectric performance in p-type PbSe

Yingcai Zhu,[1] Dongyang Wang,[1] Tao Hong,[1] Lei Hu,[2] Toshiaki Ina,[3] Shaoping Zhan,[1] Bingchao Qin,[1] Haonan Shi,[1] Lizhong Su,[1] Xiang Gao,[4] Li-Dong Zhao[1*]

[1]School of Materials Science and Engineering, Beihang University, Beijing 100191, China
[2]State Key Laboratory for Mechanical Behavior of Materials, Xi'an Jiaotong University, Xi'an 710049, China
[3]Research and Utilization Division, Japan Synchrotron Radiation Research Institute (JASRI/SPring-8), Sayo, Hyogo, Japan
[4]Center for High Pressure Science and Technology Advanced Research (HPSTAR), Beijing, 100094, China

[*]Corresponding author: zhaolidong@buaa.edu.cn (L.-D. Zhao).

Supplementary Methods

Transport properties estimation. In single parabolic band (SPB) model, the Seebeck coefficient can be expressed as:[1]

$$S = \frac{k_B}{e}\left(\frac{2F_1(\eta)}{F_0(\eta)} - \eta\right) \quad \text{(S1)}$$

where k_B is the Boltzmann constant, e is the electron charge, F_j is the Fermi-Dirac integral $F_j(\eta)=\int_0^\infty \frac{\xi^j d\xi}{1+\exp(\xi-\eta)}$, and η is the reduced Fermi level E_F/k_BT. Here, we assume that the acoustic-phonon scattering (APS) is the dominating scattering mechanism for the carriers.

Under this condition, the chemical carrier concentration (n) can be described as:

$$n=4\pi\left(\frac{2m^*k_BT}{h^2}\right)^{3/2} F_{1/2}(\eta) \quad \text{(S2)}$$

The Hall carrier concentration (n_H) is related to n via $n_H = n/r_H$, where r_H is the Hall factor and it is given by:

$$r_H = \frac{3}{4}\frac{F_{1/2}(\eta)\, F_{-1/2}(\eta)}{F_0^2(\eta)} \quad \text{(S3)}$$

For a given reduced Fermi level η, the Seebeck coefficient and Hall factor will be determined. Then, the effective mass (m^*) will be obtained via measuring n_H through Hall measurement. Therefore, the Seebeck coefficient (S) versus Hall carrier concentration (n_H) (Pisarenko plot) will be obtained with varying η for a constant m^*.

Within the APS assumption, the Hall carrier mobility (μ_H) is represented as:

$$\mu_H = \frac{e}{m^*}\frac{\pi\hbar^4 v_L^2\rho}{\sqrt{2}E_{def}^2(m^*kT)^{3/2}}\frac{F_{-1/2}(\eta)}{2F_0(\eta)} \quad \text{(S4)}$$

where v_L is the longitudinal sound velocity, ρ is the density of sample, E_{def} is the deformation potential. The E_{def} can be expressed as:

$$|E_{def}| = \left(\frac{hk_B^2}{3\pi^2}\frac{v_L^2\rho T}{m^*\kappa_L B}\right)^{1/2} \quad \text{(S5)}$$

B is the quality factor and it is given by: [2]

$$B = \left(\frac{k_B}{e}\right)^2 \frac{\sigma_{E_0} T}{\kappa_L} \tag{S6}$$

$$\sigma_{E_0} = \sigma / F_0(\eta) \tag{S7}$$

Therefore, the relation between μ_H and n_H can be estimated via equation (S1-S7).

Lorenz number calculation. The electronic contribution to the thermal conductivity was calculated via the Wiedemann-Franz law ($\kappa_e = L\sigma T$), where the Lozenz number (L) is given by:

$$L = \frac{k_B^2}{e^2}\left(\frac{3F_0(\eta)F_2(\eta) - 4{F_1}^2(\eta)}{{F_0}^2(\eta)}\right) \tag{S8}$$

Callaway model. We can estimate the lattice thermal conductivity considering the effect of defects using the Callaway model: [3]

$$\frac{\kappa_L}{\kappa_0} = \frac{\tan^{-1} u}{u} \tag{S9}$$

Here, κ_0 is the lattice conductivity of purity sample without considering defects, while the κ_L is the lattice thermal conductivity of doping material with defects. The u is a disorder parameter and it depends on the properties of pure material:

$$u^2 = \frac{(6\pi^5 V^2)^{1/3}}{2k_B v_{avg}} \kappa_0 \Gamma \tag{S10}$$

where V, v_{avg}, Γ are the average volume per atom, average speed of sound, and the disorder scattering parameter, respectively. We use the Callaway model to estimate the lattice thermal conductivity of $Pb_{0.98}Na_{0.02}Se$-x%$AgInSe_2$. To simplify, we assume the $Pb_{0.98}Na_{0.02}$ as a whole and term it as PN. Then, the chemical formula can be written as $PN_{2-2y}Ag_yIn_ySe_2$ (y = 2x/(100+2x)). Only considering the mass variation, the Γ can be expressed as:

$$\Gamma = \frac{\left[(1-y)(M_{PN} - \overline{M_1})^2 + y\left(M_{Ag} - \overline{M_1}\right)^2 + (1-y)(M_{PN} - \overline{M_2})^2 + y(M_{In} - \overline{M_1})^2\right]/4}{[(2M_{Se} + \overline{M_1} + \overline{M_2})/4]^2} \tag{S11}$$

$$M_{PN} = 0.98M_{Pb} + 0.02M_{Na} \tag{S12}$$

$$\overline{M_1} = (1-y)M_{PN} + yM_{Ag} \tag{S13}$$

$$\overline{M_2} = (1-y)M_{PN} + yM_{In} \tag{S14}$$

For a given y, the values of V, v_{avg} and κ_0 are determined as the linear interpolation between the values of the end-member species ($Pb_{0.98}Na_{0.02}Se$ and $AgInSe_2$), respectively.

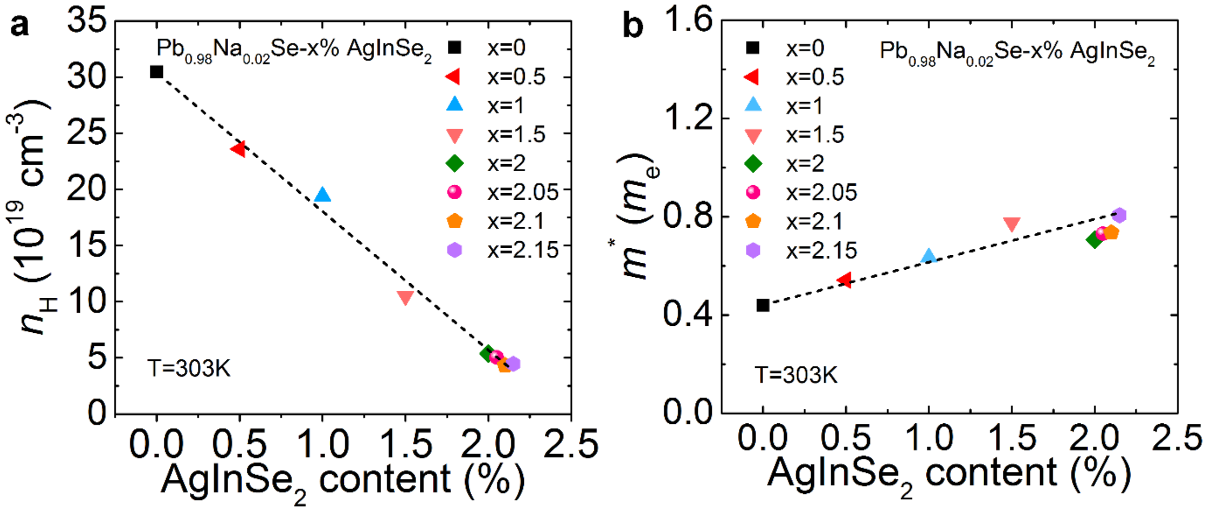

Supplementary Figure 1. (a) Hall carrier concentrations and (b) density-of-states effective mass of $Pb_{0.98}Na_{0.02}Se$ - x%$AgInSe_2$ (LISS) with increasing $AgInSe_2$ content at 303K.

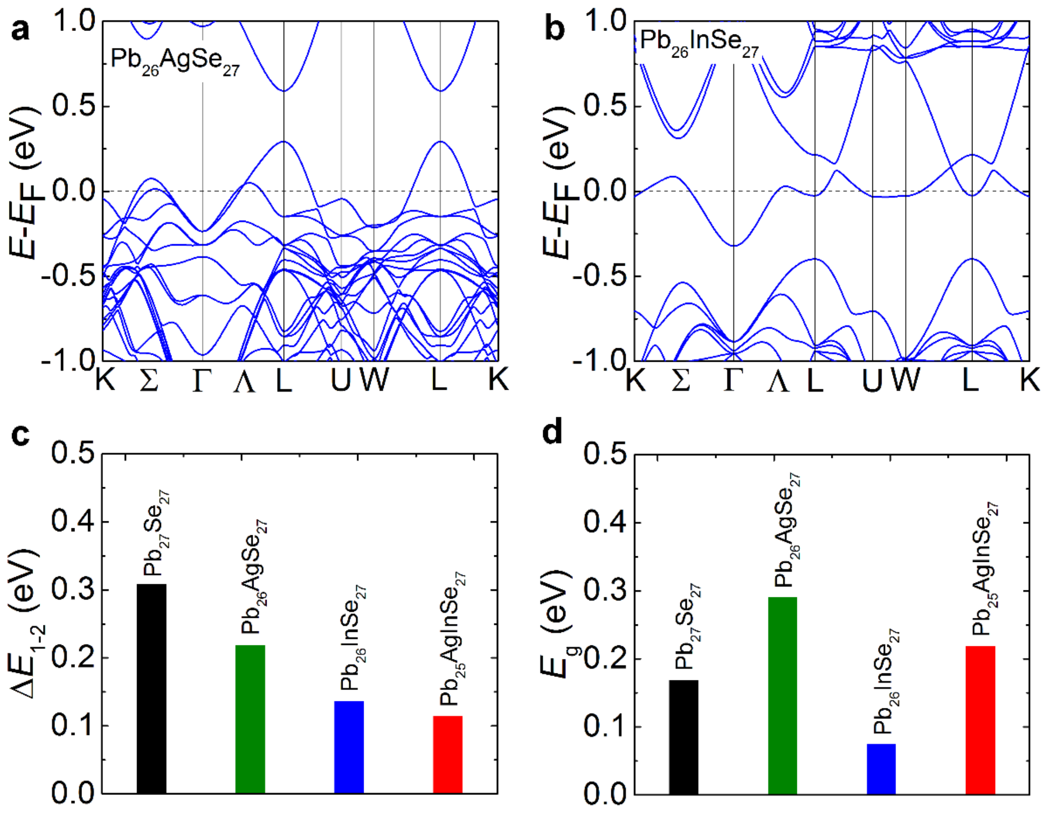

Supplementary Figure 2. Electronic band structures of (a) $Pb_{26}AgSe_{27}$ and (b) $Pb_{26}InSe_{27}$. (c) The energy offset ($\Delta E_{1\text{-}2}$) between L and Σ valence band. (d) Theoretical bandgaps (E_g) for pristine, Ag-doped, In-doped and Ag-In co-doped PbSe.

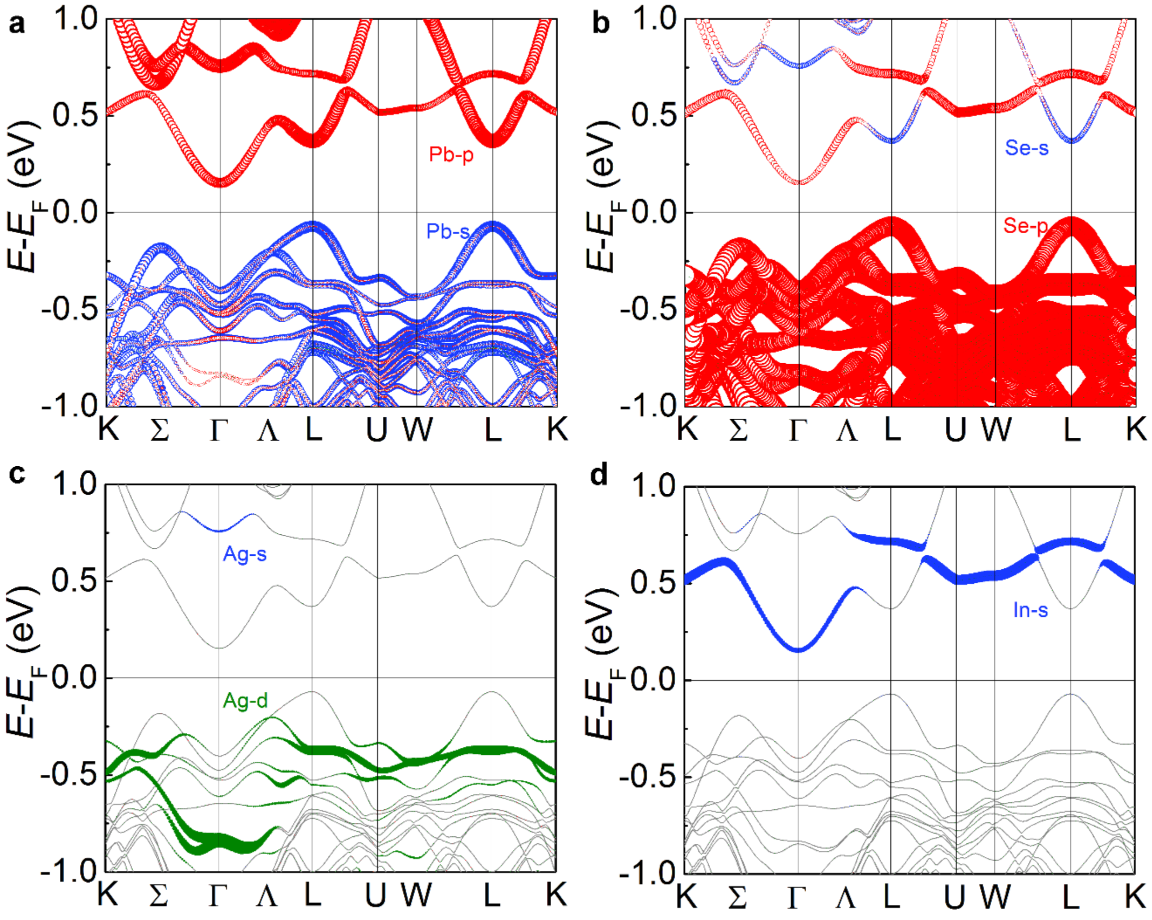

Supplementary Figure 3. The atomic orbital projected band structure of $Pb_{25}AgInSe_{27}$. (a) The conduction band is dominated by Pb-p orbitals, while the valence band contain considerable Pb-s character. (b) The Se-p orbital primarily contributes to the valence band. (c) The Ag-d orbitals have a considerable contribution to the valence band. (d) There is distinct In-s character at the conduction band.

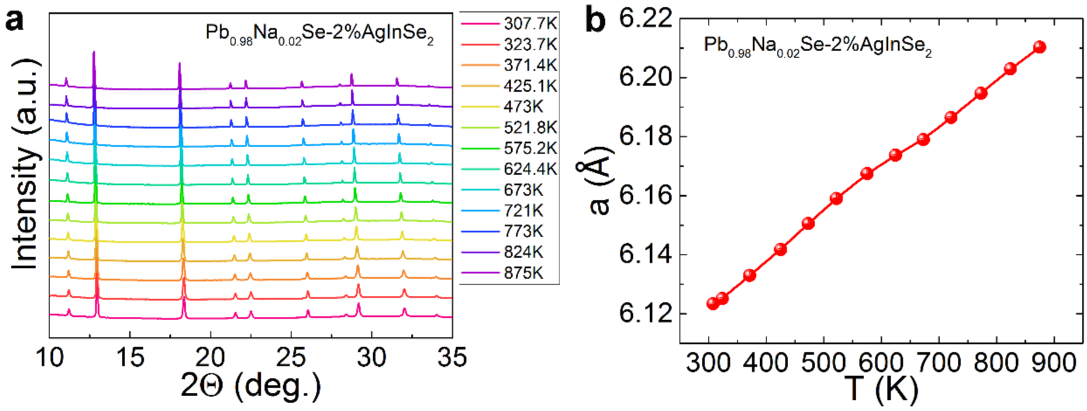

Supplementary Figure 4. (a) Temperature-dependent synchrotron radiation x-ray diffraction (SR-XRD) patterns for $Pb_{0.98}Na_{0.02}Se$-2% $AgInSe_2$. (b) The refined lattice parameters as a function of temperature.

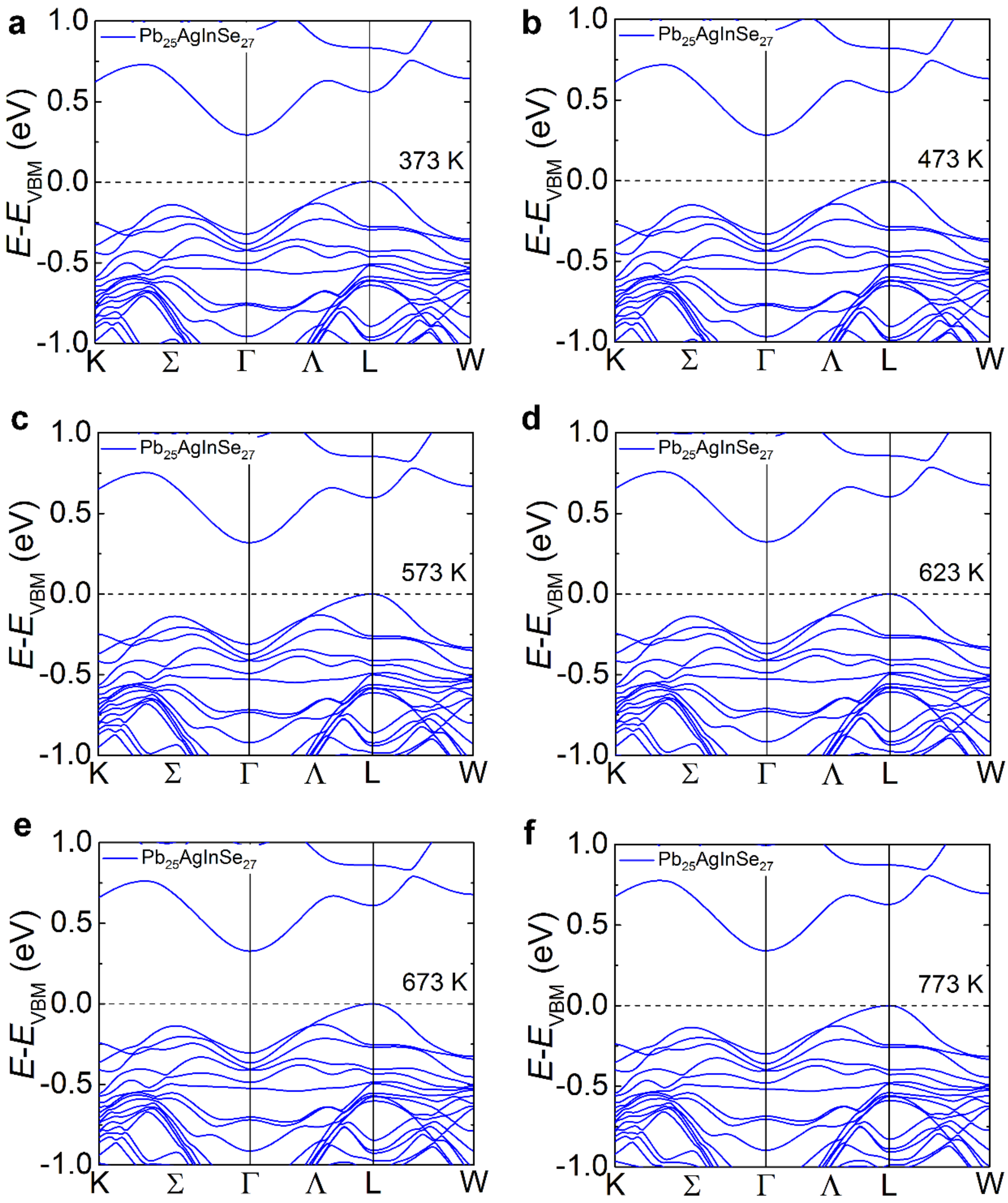

Supplementary Figure 5. The electronic band structures of $Pb_{25}AgInSe_{27}$ at various temperature: (a) 373 K, (b) 473 K, (c) 573 K, (d) 623 K, (e) 673 K, (f) 773 K.

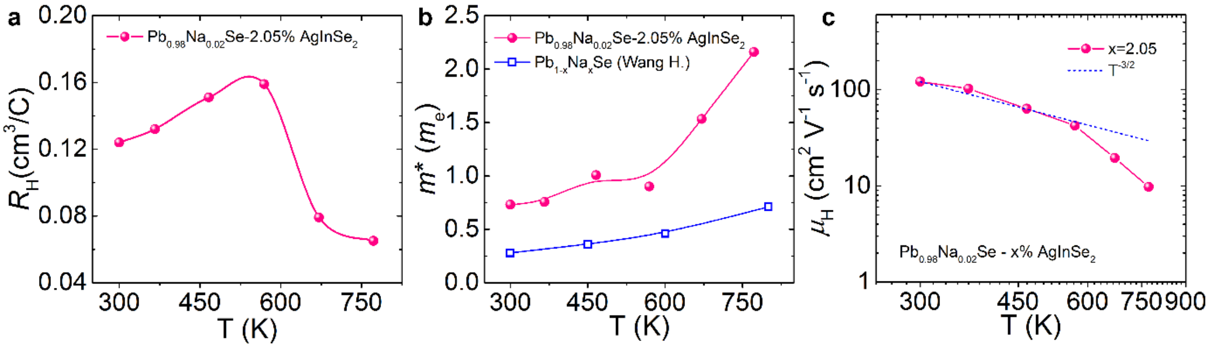

Supplementary Figure 6. (a) Temperature dependence of Hall coefficient (R_H) of $Pb_{0.98}Na_{0.02}Se$-2.05% $AgInSe_2$. (b) Effective mass as a function of temperature for $Pb_{0.98}Na_{0.02}Se$-2.05% $AgInSe_2$ and $Pb_{1-x}Na_xSe$. (c) Temperature-dependent Hall mobility of $Pb_{0.98}Na_{0.02}Se$ - 2.05% $AgInSe_2$, which displays a $T^{-3/2}$ behavior when T < 600 K, demonstrating that acoustic-phonon scattering dominates. The deviation from $T^{-3/2}$ relation at high temperature is due to the increase of effective mass.

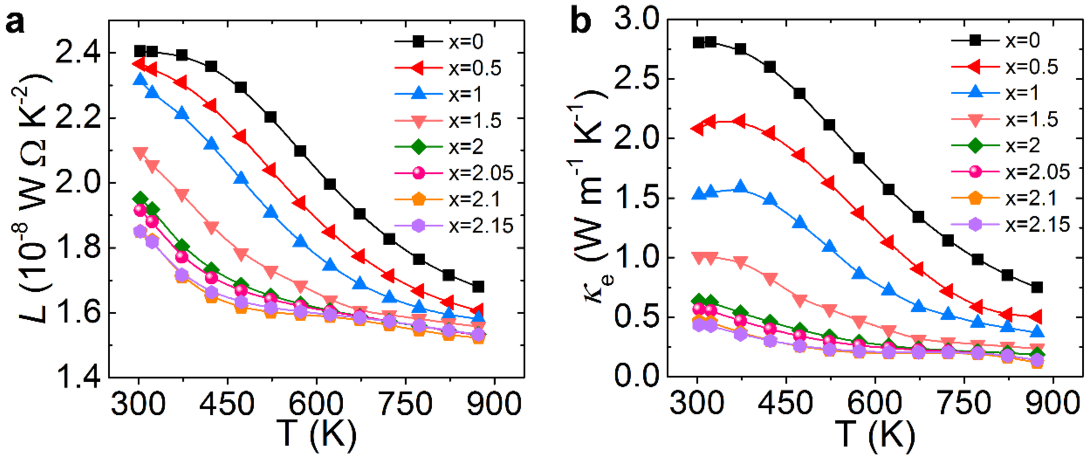

Supplementary Figure 7. Temperature-dependent (a) Lorenz number (L) and (b) electronic contribution to the thermal conductivity (κ_e) for $Pb_{0.98}Na_{0.02}Se$-x% $AgInSe_2$ (LISS).

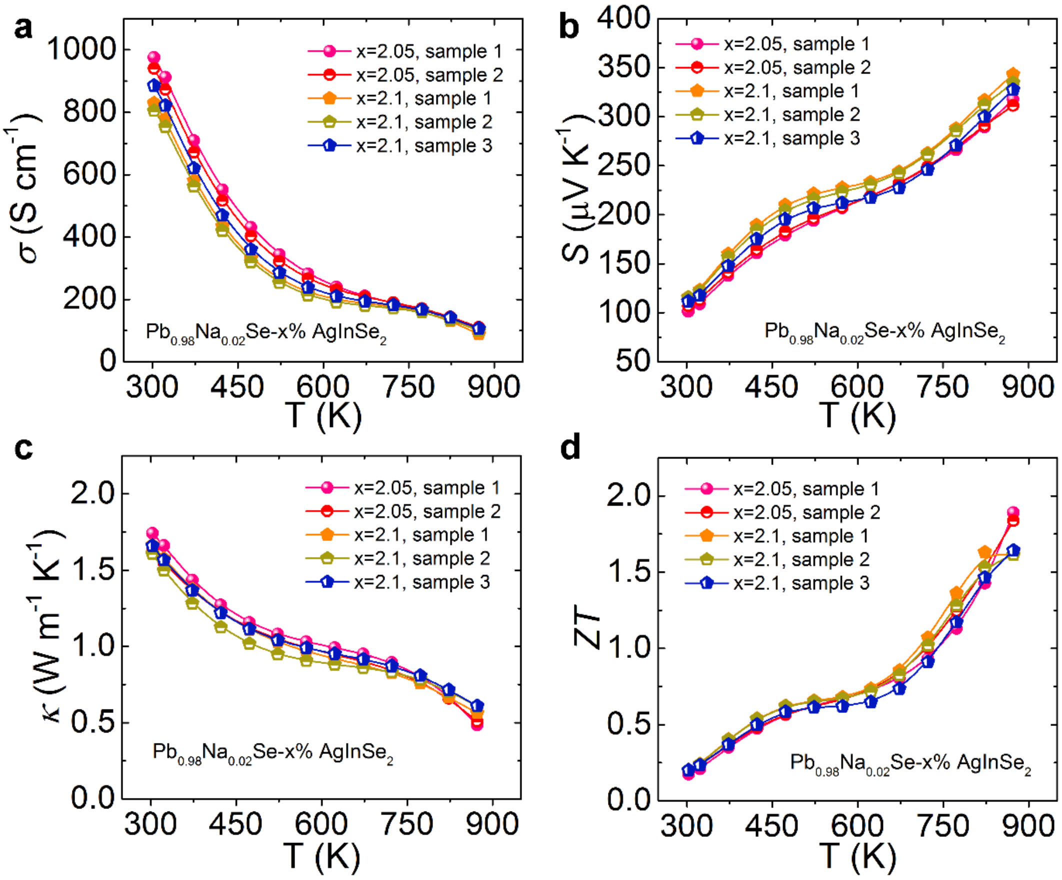

Supplementary Figure 8. Temperature-dependent (a) electrical conductivity, (b) Seebeck coefficient, (c) total thermal conductivity, and (d) figure-of-merit *ZT* for several x=2.05 and x=2.1 samples, respectively.

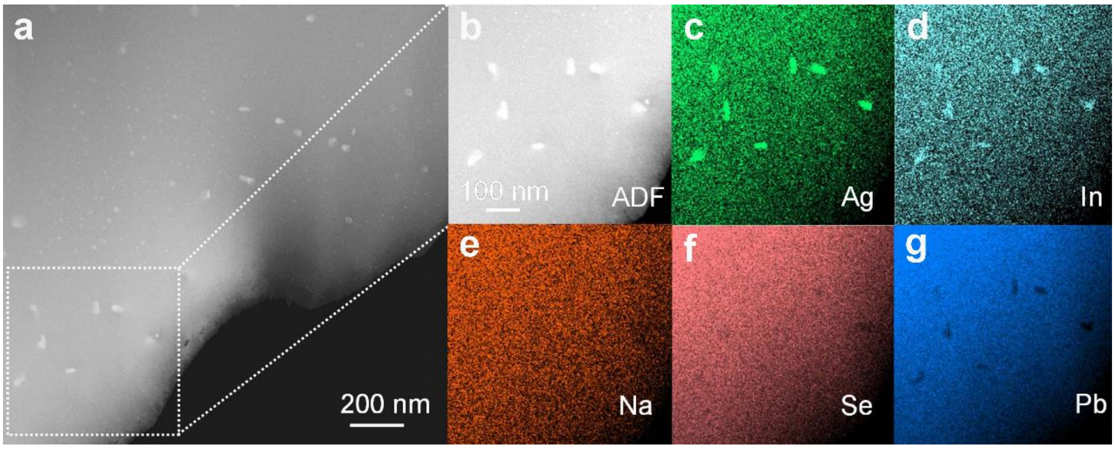

Supplementary Figure 9. Annular dark-field (ADF) STEM image (a, b) of $Pb_{0.98}Na_{0.02}Se$-2.05%$AgInSe_2$ sample and the corresponding energy dispersive X-ray spectroscopy (EDS) elements distribution mappings for (c) Ag, (d) In, (e) Na, (f) Se and (g) Pb elements, respectively.

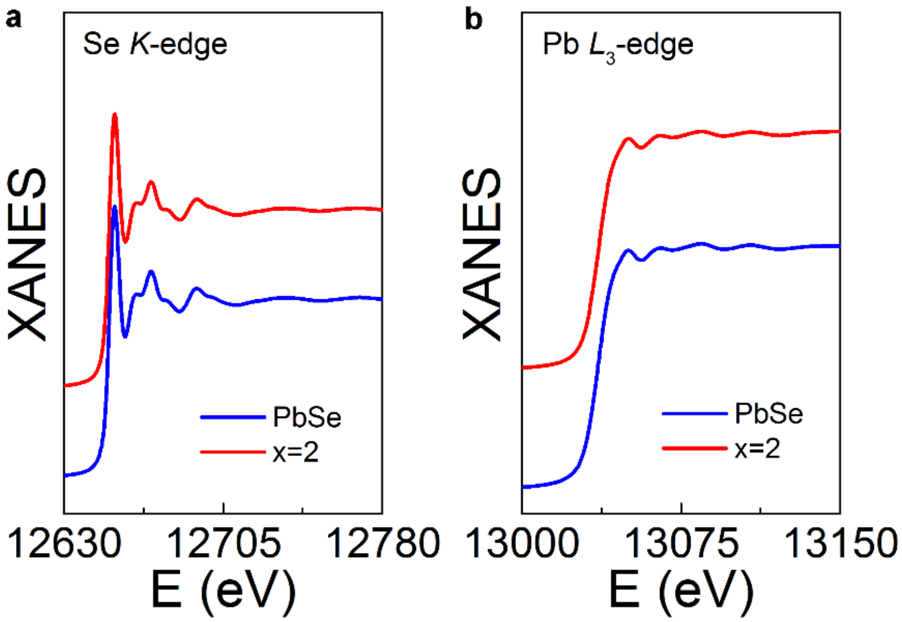

Supplementary Figure 10. Comparison of experimental XANES spectra of (a) Se *K*-edge and (b) Pb L_3-edge between PbSe and $Pb_{0.98}Na_{0.02}Se$-2%$AgInSe_2$.

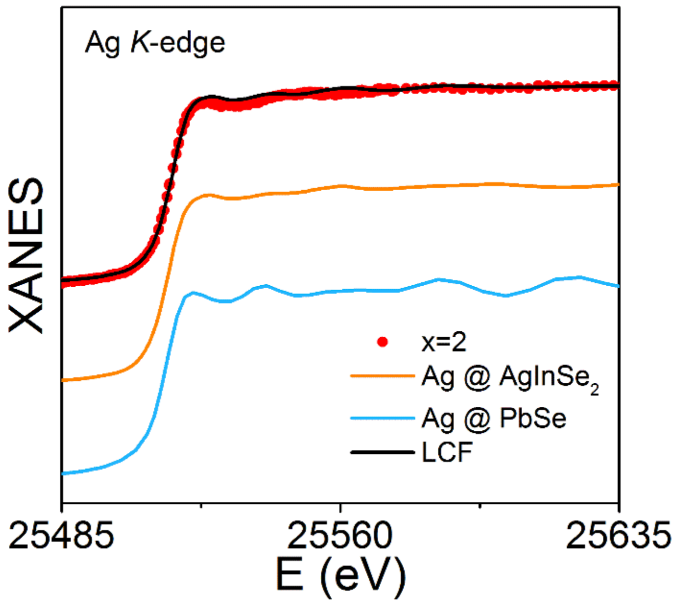

Supplementary Figure 11. Experimental XANES spectra of Ag *K*-edge for $Pb_{0.98}Na_{0.02}Se$-2% $AgInSe_2$ (red dots), and $AgInSe_2$ (orange line), respectively. Theoretical XANES spectrum of Ag *K*-edge for Ag-doped PbSe assuming that Ag occupy the Pb site. The black line represents a linear combination fitting result of Ag *K*-edge of $Pb_{0.98}Na_{0.02}Se$-2% $AgInSe_2$ considering that the Ag *K*-edge of $AgInSe_2$ and Ag-doped PbSe serves as standards.

Supplementary Table 1. Various parameters (Longitudinal sound velocity (v_l), transverse sound velocity (v_t), average sound velocity (v_{avg}), Poisson ration (v_p), Grüneisen parameter (γ), and bulk modulus (K)) of $Pb_{0.98}Na_{0.02}Se$ - x% $AgInSe_2$. The Poisson ration (v_p) is calculated by $v_p = \frac{1-2(v_t/v_l)^2}{2-2(v_t/v_l)^2}$, the Grüneisen parameter (γ) is obtained using $\gamma = \frac{3}{2}\left(\frac{1+v_p}{2-3v_p}\right)$ and the bulk modulus (K) is given by $K = \rho\left(v_l^2 - \frac{4}{3}v_t^2\right)$ (ρ is the density of sample).

Sample	v_l (m/s)	v_t (m/s)	v_{avg} (m/s)	v_p	γ	K (GPa)
x=0	3165.6	1708.6	1907.1	0.294	1.74	48.9
x=0.5	3192.9	1726.9	1927.3	0.293	1.73	50.2
x=1	3214.7	1726.1	1927.4	0.297	1.75	51.2
x=1.5	3151.7	1715.4	1913.5	0.289	1.71	48.0
x=2	3217.4	1720.9	1922.1	0.299	1.77	50.9
x=2.05	3148.7	1718.3	1916.4	0.288	1.70	47.6
x=2.1	3179.8	1720.3	1919.9	0.293	1.73	48.3
x=2.15	3149.7	1718.8	1916.9	0.288	1.70	47.4

Supplementary Table 2. LCF fitting results of In K-edge and Ag K-edge of $Pb_{0.98}Na_{0.02}Se$ - 2% $AgInSe_2$.

Atom	Atomic occupied ratio in standards	
	@ Pb site in PbSe	$AgInSe_2$
In	13.7%	86.3%
Ag	16.0%	84.0%

Supplementary References

1 May, A. F. & Snyder, G. J. Thermoelectric Handbook: Thermoelectrics and its Energy Harvesting. *CRC Press: Boca Raton, FL* **Vol. 1**, pp 231-248 (2012).

2 Kang, S. D., Snyder, G. J. Charge-transport model for conducting polymers. *Nat. Mater.* **16**, 252-257 (2017).

3 Joseph, C. & Hans C., v. B. Effect of Point Imperfections on Lattice Thermal Conductivity. *Phys. Rev.* **120**, 1149-1154 (1960).
